# Supplementary material for: Targeting serum phosphate trajectory stratification to improve outcomes in high-risk Cardiovascular-Kidney-Metabolic-Sepsis cohorts
Source: PLoS One. 2025 Aug 21;20(8):e0330497. doi: 10.1371/journal.pone.0330497 (PMC12370140; doi:10.1371/journal.pone.0330497)
Supplement: S1 Fig. S1 — This figure presents the results of consensus clustering analysis to determine the optimal number of clusters (k) for Cardiovascular-Kidney-Metabolic-Sepsis patients. (A) Delta area plot: Shows the relative change in the area under the cumulative distribution function (CDF) curve as k increases, indicating the optimal number of clusters. (B) Consensus CDF plot: Displays the cumulative distribution function (CDF) for different k-values, where greater separation and stability of CDF curves suggest the most suitable cluster number. (DOCX) [file pone.0330497.s001.docx]

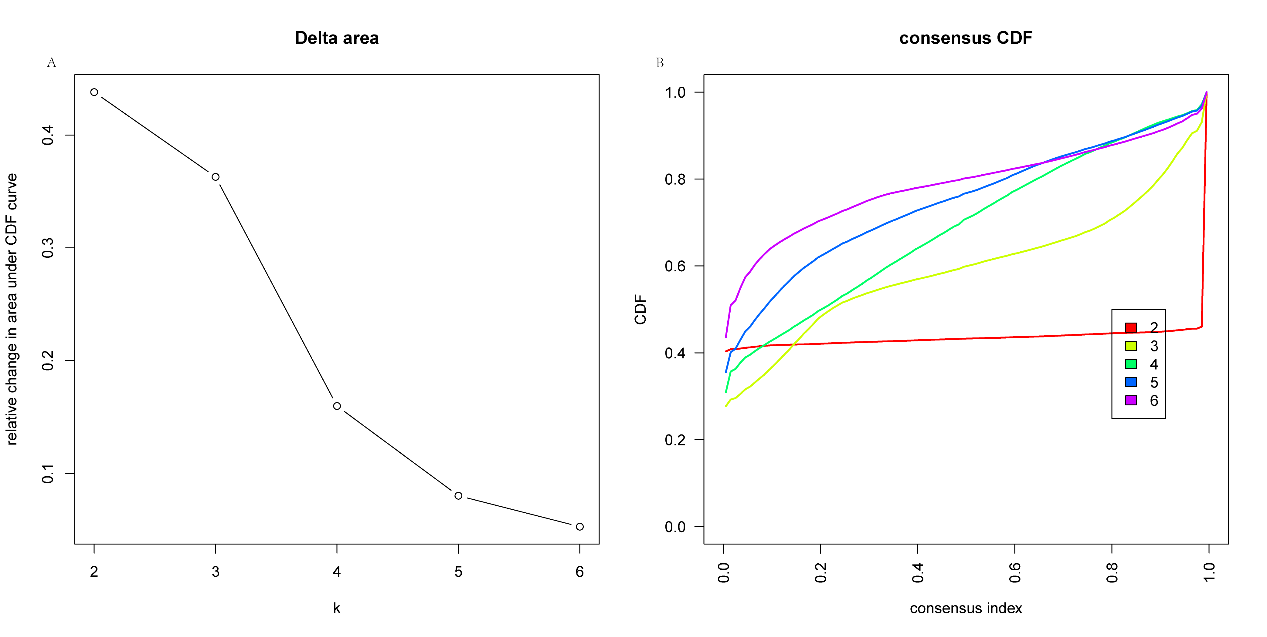


**Fig S1**：**K-means Clustering Classification Patients**
This figure presents the results of consensus clustering analysis to determine the optimal number of clusters (k) for Cardiovascular-Kidney-Metabolic-Sepsis patients.
(A) Delta area plot: Shows the relative change in the area under the cumulative distribution function (CDF) curve as k increases, indicating the optimal number of clusters.
(B) Consensus CDF plot: Displays the cumulative distribution function (CDF) for different k-values, where greater separation and stability of CDF curves suggest the most suitable cluster number.
