# Supplementary figures and images for: Targeting serum phosphate trajectory stratification to improve outcomes in high-risk Cardiovascular-Kidney-Metabolic-Sepsis cohorts
Source: PLoS One. 2025 Aug 21;20(8):e0330497. doi: 10.1371/journal.pone.0330497 (PMC12370140; doi:10.1371/journal.pone.0330497)

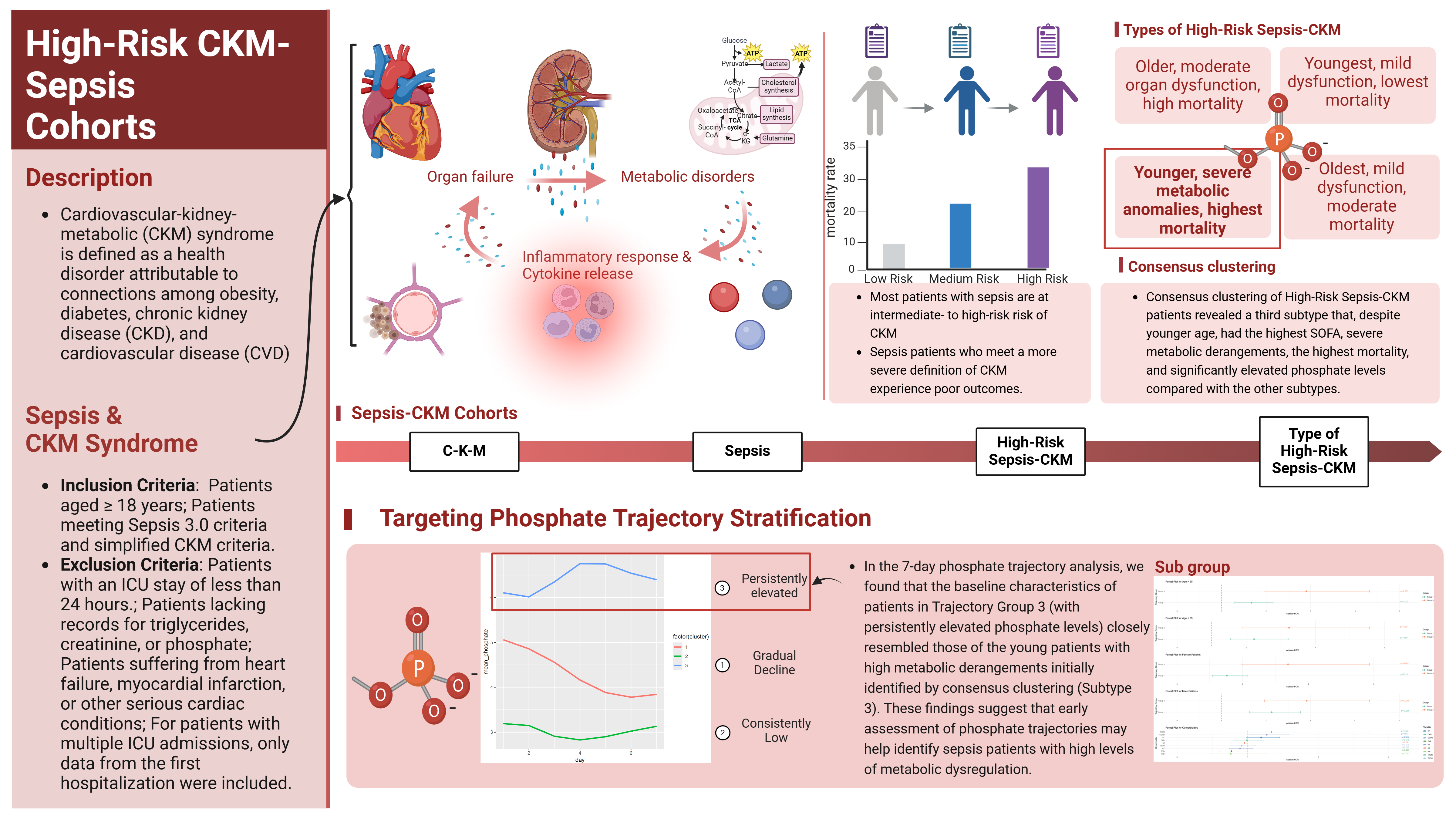

Supplement: S2 Graphic abstract — This schematic illustrates the interplay between cardiovascular–kidney–metabolic (CKM) syndrome and sepsis, highlighting patient stratification from lower to higher risk, potential clinical characteristics, and therapeutic targets. It underscores the importance of phosphate metabolism in disease progression and prognosis. (PNG) [file pone.0330497.s002.png]
