# Supplementary material for: Targeting serum phosphate trajectory stratification to improve outcomes in high-risk Cardiovascular-Kidney-Metabolic-Sepsis cohorts
Source: PLoS One. 2025 Aug 21;20(8):e0330497. doi: 10.1371/journal.pone.0330497 (PMC12370140; doi:10.1371/journal.pone.0330497)
Supplement: Attachment — Submitted filename: Response to reviewers.docx [file pone.0330497.s004.docx]

**Response to Reviewers**

**Manuscript ID:** PONE-D-25-14898R1
**Title:** *Targeting Serum Phosphate Trajectory Stratification to Improve Outcomes in High-Risk Cardiovascular-Kidney-Metabolic-Sepsis Cohorts*

Dear Academic Editor and Reviewers,

We sincerely thank you for your constructive feedback, which greatly improved the quality and clarity of our manuscript. Below we provide a point-by-point response to the remaining comment from Reviewer #1. All revisions are clearly highlighted in the tracked-changes version of the manuscript.

**Reviewer #1**

**Comment:**
Thank you for your responses. There is still one remaining issue that needs clarifying. I asked you to talk about the association between phosphate metabolism and CKM pathophysiology; however, due to the complexity of CKM pathophysiology, you did not add details regarding this issue. Try to utilize studies which evaluated this unclear and complex pathophysiology and expand it in the introduction or discussion.

**Response:**
Thank you for this important comment. We agree that the role of phosphate metabolism in CKM pathophysiology deserves further elaboration. In the revised manuscript, we have added a new paragraph to the Introduction to describe how dysregulated phosphate metabolism contributes to vascular calcification, endothelial dysfunction, insulin resistance, and the progression of CKM-related disorders. Page 4, line 86-94. We also integrated this discussion further in the Discussion section, highlighting its implications for systemic metabolic stress, particularly in high-risk sepsis populations. Page 14-15, line 345-348.

To support these additions, we have cited the following key references:

- Ndumele et al. (2023), *Circulation*: to contextualize CKM as an emerging clinical framework.
- Raikou (2021), *World J Nephrol*: to support the link between serum phosphate and cardiorenal pathology.
- Nakanishi et al. (2020), *Free Radic Biol Med*: to describe the pathological role of phosphate and FGF23 in CKD and cardiovascular complications.

We trust that these revisions now adequately address your insightful suggestion.

Sincerely,
Zhihong Zuo, PhD
On behalf of all co-authors
